# A Rapid Review on the Effectiveness and Use of Wearable Biofeedback Motion Capture Systems in Ergonomics to Mitigate Adverse Postures and Movements of the Upper Body

**DOI:** 10.3390/s24113345

**Published:** 2024-05-23

**Authors:** Carl M. Lind

**Affiliations:** IMM Institute of Environmental Medicine, Karolinska Institutet, 171 77 Stockholm, Sweden; carl.lind@ki.se

**Keywords:** ergonomics, intervention, review, work postures, musculoskeletal disorders, musculoskeletal pain, wearables, biofeedback, augmented feedback, work technique

## Abstract

Work-related diseases and disorders remain a significant global health concern, necessitating multifaceted measures for mitigation. One potential measure is work technique training utilizing augmented feedback through wearable motion capture systems. However, there exists a research gap regarding its current effectiveness in both real work environments and controlled settings, as well as its ability to reduce postural exposure and retention effects over short, medium, and long durations. A rapid review was conducted, utilizing two databases and three previous literature reviews to identify relevant studies published within the last twenty years, including recent literature up to the end of 2023. Sixteen studies met the inclusion criteria, of which 14 were of high or moderate quality. These studies were summarized descriptively, and the strength of evidence was assessed. Among the included studies, six were rated as high quality, while eight were considered moderate quality. Notably, the reporting of participation rates, blinding of assessors, and a-priori power calculations were infrequently performed. Four studies were conducted in real work environments, while ten were conducted in controlled settings. Vibration feedback was the most common feedback type utilized (n = 9), followed by auditory (n = 7) and visual feedback (n = 1). All studies employed corrective feedback initiated by the system. In controlled environments, evidence regarding the effectiveness of augmented feedback from wearable motion capture systems to reduce postural exposure ranged from strong evidence to no evidence, depending on the time elapsed after feedback administration. Conversely, for studies conducted in real work environments, the evidence ranged from very limited evidence to no evidence. Future reach needs are identified and discussed.

## 1. Introduction

### 1.1. Work-Related Diseases and Disorders

Work-related diseases and disorders remain a significant global health concern, with an estimated 1.7 billion people worldwide experiencing adverse musculoskeletal health conditions [1]. The primary categories of musculoskeletal disorders (MSDs) are low back pain and neck pain. In 2005, low back pain and neck pain ranked as the eighth most common cause of disability-adjusted life years globally. By 2015, they had risen to the fourth most common cause, accounting for an estimated 95 million disability-adjusted life years worldwide [2]. Occupational accidents, diseases, and musculoskeletal disorders account for approximately 40% of global compensation costs [3] with an estimated cost of 3.3% of the gross domestic product in the European Union and 3.9% globally [4].

Work-related MSD (WMSD) encompass injuries and illnesses of the locomotor apparatus caused, aggravated, accelerated, or exacerbated by interaction with known or unknown factors in work activities [5,6,7]. The causes of WMSDs are multifactorial and comprise physical and psychosocial risk factors [6,8,9]. In addition to hand-arm and whole-body vibrations [6,10,11,12], major work-related physical risk factors include heavy and repetitive manual handling [6,10,13,14,15,16,17,18,19,20] and awkward postures and rapid movements of primarily the upper body [6,10,14,20,21,22,23,24]. These risk factors are prevalent in the working population. For example, according to self-reported data [25], about six out of ten workers in the European Union engage in repetitive hand and arm movements at least one-quarter of their worktime, and around three out of ten perform such movements all or most of the time. Close to half are exposed to tiring and painful work postures at least one-quarter of the worktime, and 14% are exposed to such postures all or most of the time. In addition to causing years without disability and imposing high costs on both individuals and society, WMSDs can impair work capacity and increase the risk of short- and long-term absenteeism and premature exit from the labor market [26,27,28]. Given the widespread occurrence of WMSDs, proactive measures are necessary to prevent ill health conditions. These actions include screening and risk assessment of hazards as part of systematic risk management [29,30,31], often conducted with the assistance of observation-based tools [32,33,34,35,36,37]. While these tools can facilitate the assessment of a wide range of risk factors, they sometimes lack accuracy and precision, particularly in estimating exposure to distal upper limb risks [38,39,40,41,42,43,44]. Therefore, technical measurement instruments can be used as a complement to enhance the accuracy and precision of the measurements [45].

### 1.2. Work Technique Training

Effective risk-reducing measures should typically adhere to the hierarchy of control, prioritizing the elimination of hazards as the primary action, followed by the substitution of tasks, processes, and substances. Additional measures include engineering and administrative controls, with the implementation of personal protective equipment as a last option. Examples of engineering and administrative controls to target WMSDs include, e.g., the organization of work (staffing, training or workers, and schedules), the re-arrangement of workspaces, and the introduction of new or optimized work equipment. 

In manual handling tasks, automation or the introduction of lift equipment are commonly employed measures [46,47,48]. Employee training often encompasses the safe operation of work equipment and techniques to minimize adverse postures or movements that can lead to increased biomechanical stress, such as reducing excessive bending and twisting of the trunk. Traditional work technique training targeting WMSD risks typically involves theoretical education on risk factors and practical training using a few simulated work tasks, for a short period and in a controlled environment, rather than tasks in real work situations. However, recent systematic reviews suggest that traditional work technique training has little to no clinically relevant effects, raising questions about its effectiveness in reducing WMSDs [49,50,51,52]. Several factors have been proposed to enhance effectiveness, including conducting training sessions with more realistic work tasks instead of simplified ones that represent only a fraction of actual work tasks. Additionally, extending the training period to promote motor learning and thereby facilitate lasting behavioral changes has been recommended [52].

### 1.3. Sensor-Based Training

The use of sensor-based systems with augmented feedback offers a potential solution to support training in more complex environments, including real work contexts, over longer periods, and with direct feedback to each employee. Compared to ocular or visual observation, sensor-based instruments and systems can detect smaller differences in postures and movements due to their higher accuracy and precision [53]. Additionally, these systems do not require the presence of an observer, such as an instructor [54]. In contrast to video-based systems [55,56], which may be applicable in non-ambulatory work contexts, ambulatory sensor-based instruments and systems can be used for both ambulatory and non-ambulatory work situations. Sensor-based instruments and systems have been utilized across various contexts to improve movements and task execution of both the upper and lower body, including rehabilitation, sports, and ergonomics [45,57,58,59,60,61,62,63,64,65,66,67]. In the field of ergonomics, research on the effectiveness of augmented feedback from wearable motion capture sensor systems to reduce adverse postures and movements has primarily emerged in the last 10–20 years [45,66,67]. Recent overviews and reviews indicate that feedback typically involves audio, vibration, or visual feedback, or a combination of the three [45,66,67,68,69].

Lee et al. [66] reviewed 14 studies, of which 11 papers included at least eight participants receiving feedback, and 12 studies published in peer-reviewed journal articles. They concluded that there was limited evidence that augmented feedback from wearables reduces adverse postures of the neck, upper arms, and trunk, as well as pain or discomfort of the neck and lower back. The inclusion of studies with a small population, such as those with fewer than eight subjects, may increase the risk of type-II error (false-negative), especially if the sample size has not been justified based on statistical power calculations. Additionally, only a few of the included studies were conducted in real contexts or contexts that more closely represent real work tasks. Additionally, there were a few studies published at the time seemingly fitting the inclusion criteria that were not included in the review (i.e., Owlia et al. [70]; Lind et al. [71]; Ribeiro et al. [72]; and Kuo et al. [73]).

Frasie et al. [67] reviewed the evidence regarding the effectiveness of extrinsic feedback for preventing and rehabilitating WMSDs, focusing on its effects on function (e.g., work ability and disabilities), symptoms (e.g., discomfort and pain), and sensorimotor control (e.g., posture and muscle activation). Among the 49 included studies, 12 targeted sensorimotor control in controlled environments, while another 12 focused on real work environments. The studies evaluated various feedback types including verbal feedback from instructors and wearable extrinsic feedback from IMUs and sEMG instruments and systems, as well as non-wearable instruments and systems, such as vibration feedback provided by pressure sensors in chairs. They concluded that there was moderate evidence supporting the effectiveness of feedback in controlled environments, while the evidence in real work environments was conflicting. However, the studies encompassed the rehabilitation of patients with special conditions, as well as a mix of work-related and non-work-related tasks, and both ambulatory and non-ambulatory instruments and systems, as well as verbal feedback from instructors. This limits the generalizability of the findings to broader work populations performing regular work-related tasks. Notably, some potentially relevant studies were not included in the review (e.g., Doss et al. [74] and Lind et al. [71]) and since then, several additional studies have been published (e.g., Lim et al. [75], Langenskiöld et al. [76], and Lind et al. [77]). Lind et al. [45] reviewed the literature on wearable motion capture instruments and systems, exploring their potential applications for preventing WMSDs and proposing a taxonomy for classifying augmented feedback. However, their review did not systematically evaluate the evidence regarding the effectiveness of these systems in modifying postures or movements, nor did it assess the methodological quality of the studies or the strength of the evidence.

Subsequently, two additional reviews on augmented feedback have been published [68,69]. The systematic review by García-Jaén et al. [68] included eight studies focusing on the lumbar spine. The review encompassed studies involving both healthy participants and participants with more chronic (long-term) conditions and assessed the methodological quality of each study. It provided a comprehensive synthesis of the literature, including the instrumentation utilized, types of feedback administered, and practical applications. While acknowledging the need for further investigations, particularly long-term studies, they did not systematically assess the strength of evidence of the effectiveness of biofeedback and omitted several studies that examined feedback to improve back postures, which were included in the overview by Lind et al. [45] (e.g., Doss et al. [74] and Lind et al. [71,77]). Similarly, Figueira et al.’s review [69], comprising twelve articles, provided an overview of the literature regarding feedback modalities, targeted anatomical regions, and workplace settings. However, it did not undertake an assessment of methodological quality or the strength of evidence pertaining to the efficacy of biofeedback in mitigating adverse postures or other outcomes. Furthermore, this review omitted several relevant studies included in Lind et al.’s overview [45] (e.g., Lind et al. [78], Owlia et al. [70], and Kamachi et al. [79]).

### 1.4. Research Gap

Based on the existing reviews, there is a gap in the literature regarding the efficacy of augmented feedback from wearable motion capture systems in mitigating adverse postures and movements, particularly considering the latest research. In this context, wearables devices refers to “gadgets, accessories, or clothes with incorporated self-powered electronics and software that are capable of sensing, processing, and storing, and have communication capabilities that can be comfortably worn on the human body or be implanted on or under the skin, and that are not perceived as obtrusive and hindering performance (such as work performance)” [45]. Current studies encompass a mixture of laboratory-based studies and studies conducted in real work environments. Some studies assess effectiveness during or shortly after feedback application, while others evaluate it over days, weeks, or months. It is hypothesized that the effectiveness of augmented feedback may vary between controlled, less complex settings (e.g., laboratory experiments) compared to when applied in real work environments. Furthermore, it is suggested that augmented feedback may be more effective in the short term and its effectiveness may gradually fade over time. However, these hypotheses have not been fully explored in current reviews. Hence, there is a need to assess effectiveness separately in each of these scenarios.

### 1.5. Aim

The aim of this rapid review is to address the identified research gap by assessing the effectiveness of wearable motion capture sensor systems that utilize augmented feedback to mitigate adverse work-related postures and movements of the upper body. The assessment of its effectiveness includes temporal aspects ranging for immediate feedback (i.e., during and initially after the feedback is provided) to short, medium, and longer-term effects (i.e., retention effects) and separately for controlled environments (non-work context) and real work contexts.

## 2. Methods

Given the fast-growing research area, a rapid review was found appropriate giving its quicker process compared to systematic literature reviews [80]. The guidelines for rapid reviews by the Cochrane Rapid Reviews Methods Group [80] were applied, and the articles were structured using the PRISMA 2020 guideline [81].

### 2.1. Eligibility Criteria 

Peer-reviewed journal articles written in English were included if they presented results from an evaluation of augmented feedback from wearables targeting postures and/or movements of the upper body in work-related activities. Application in sports and rehabilitation are excluded (Table 1). 

### 2.2. Search Strategy

**Figure 1:**
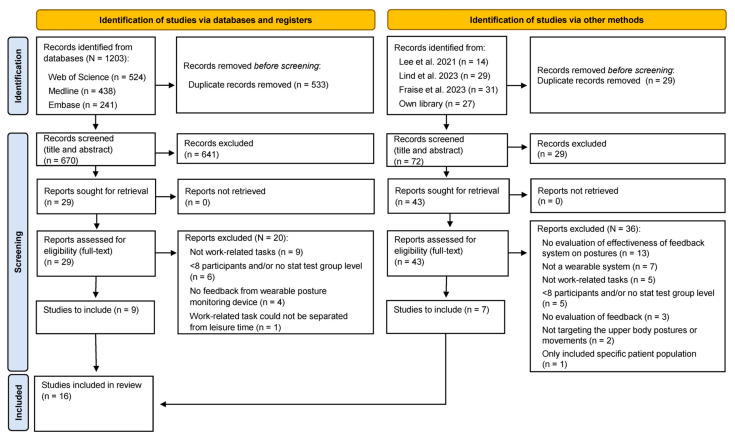
PRISMA 2020 flow diagram of the study inclusion process [45,66,67].

To identify literature, a systematic electronic literature search was performed using three databases: Web of Science, Medline and Embase (Figure 1). 

The search period for the first two databases ranged from 1 January 2020, to 30 November 2023, while for Embase, it extended from 1 January 2020, to 1 December 2023 (refer to Appendix A for the search strings used). In addition to this, older literature (i.e., before 2020) was identified through recent systematic reviews by Lee et al. [66], Lind et al. [45], and Frasie et al. 2023 [67], of which the study by Lee et al. [66] covered literature from 2005 to 15 July 2021, and Frasie et al. 2023 [67] included studies from 1986. Further, reference lists of included articles and personal libraries of the author were screened for additional relevant articles. The search was conducted between 30 November 2023, and 1 December 2023. Duplicate records were removed using the “Remove Duplicates” function in Microsoft 365 Excel (Microsoft Corporation, Redmond, WA, USA), followed by manual verification for any remaining duplicates.

### 2.3. Study Selection

One reviewer (C.M.L.) screened the identified articles and applied the inclusion criteria based on the titles and abstracts of each article (Figure 1). For articles where the assessment of the inclusion criteria could not be determined from the titles and abstracts alone, the full-text articles were retrieved and assessed against the inclusion criteria. If it remained unclear whether an article met the inclusion criteria after reviewing the full text, a second reviewer was consulted for further assessment.

### 2.4. Data Extraction

The main characteristics of the included studies were extracted by one reviewer (C.M.L) and included the following:

- Study objective and design, including the utilization of a comparison or control group.
- Description of study settings and tasks performed.
- Participant details, including the count, level of experience, gender, age, body mass, stature, and inclusion criteria.
- Duration of feedback follow-up and description of conditions or sessions, such as baseline and feedback conditions.
- Characteristics of the feedback, categorized based on the feedback taxonomy [45], including type, modality, initiation, and timing.
- Threshold for initiating feedback (feedback trigger) based on exposure.
- Motion capture systems and instruments utilized, including the type of device and its position on the body.
- Systems and instruments used for analyzing motion capture data and providing feedback, including device type and position on the body.
- Level of wearability of the systems and instruments.
- The results of each study (where proportional differences were extracted, or calculated if not provided by the original source).

Predetermined outcomes, shown in Table 2, were used to evaluate the evidence of an effect of the feedback. 

### 2.5. Risk of Bias Assessment/Methodological Quality Assessment

In accordance with the Cochrane recommendations for rapid reviews [80], one reviewer first assessed each item for its methodological quality (risk of bias). 

The methodological quality was assessed using the study quality assessment tools developed by the National Heart, Lung, and Blood Institute [82] for the assessment of interventions studies. They comprise five different tools depending on the study design and have been used in several recent reviews of ergonomics interventions (e.g., [66,83]). For this study, the tool to assess controlled intervention studies, and the tool to assess observational cohort and cross-sectional studies were used (see Appendix B, Table A3 and Table A4). 

Based on the previous reviews [45,66,67], it was assumed that the majority of identified studies could be assessed using the tool for observational cohort and cross-sectional studies. Considering that the use of different tools can influence the final quality rating, it was decided, prior to analysis, to minimize the number of different tools applied. For studies not primarily falling under observational cohort or cross-sectional designs (e.g., RCT studies), both quality-assessment tools were applied.

Each study was assessed as to whether it fulfilled each criterion categorized as follows:

- Fulfilling the criterion,
- Not fulfilling the criterion,
- Not reported, i.e., no information could be retrieved to answer the question,
- Not applicable, i.e., the criterion was judged as not applicable for the study design.

A score of one was assigned to each criterion that was fulfilled (yes), while a null score was assigned to a criterion that was not fulfilled (no) or could not be identified (not reported). Criteria judged as not applicable were excluded from the total sum score, resulting in an individual sum score for each study.

To classify the level of methodological quality, studies with a sum score of ≥75% (positive-item checks) were rated as high quality, those with a sum score of 50–74% were rated as moderate quality, and those with a sum score of <50% were rated as low quality. Only studies rated as high and moderate quality were included. For studies where two tools were used to assess methodological quality, it was pre-decided to include a study if either quality assessment tool indicated a moderate or high-quality rating.

### 2.6. Strength of Eavidence Assessment

The strength of the evidence was assessed based on the framework provided by Lee et al. [66], which categorizes the strength into seven levels ranging from strong evidence to no evidence (Table 3). The assessment was conducted by a single reviewer (C.M.L.). 

## 3. Results

A total of 1304 records were initially identified from the three databases and additional sources (Figure 1). After removing duplicates (n = 592), 670 records underwent screening based on their titles and abstracts. Subsequently, 72 records underwent full-text screening, and upon applying the inclusion criteria, 16 peer-reviewed articles were identified. Each of these articles encompassed one unique and relevant study.

### 3.1. Quality Assessment

The methodological quality of the 16 studies was evaluated, as outlined in Table 4 and Table 5. Upon assessment, six studies (38%) were rated as high-quality, while eight studies (50%) were categorized as moderate quality. Additionally, two studies (13%) were excluded from the review due to being rated as low quality. For two studies [54,72], the methodological quality was assessed using two tools each, both resulting in the same quality category rating. Among the initial 16 studies assessed, the criteria least fulfilled were as follows:

- Reporting the participation rate of eligible persons (not fulfilling the criterion, n = 15)
- Blinding of assessors (not fulfilling the criterion, n = 15)
- Reporting a priori statistical power calculation (not fulfilling the criterion, n = 12).

**Table 4:** Methodological quality assessment based on the NHLBI observational cohort or cross-sectional studies tool.

Study	Criteria													Rating	
	1	2	3	4	5	6	7	8	9	10	11	12	13	14	
Ailneni et al. [84]	+	+	NR	NA	NR	NA	+	+	−	+	+	NR	+	−	MQ
Bazazan et al. [85]	+	+	NR	+	NR	+	+	+	−	+	−	NR	+	−	MQ
Boocock et al. [86]	+	+	NR	+	+	NA	+	+	+	+	+	NR	+	+	HQ
Bootsman et al. [87]	+	+	NR	NA	NR	+	+	+	−	+	+	NR	+	−	MQ
Doss et al. [74]	+	+	NR	NA	NR	+	+	+	−	+	+	NR	+	−	MQ
Kamachi et al. [79]	+	+	NR	+	+	+	+	+	+	+	+	NR	+	+	HQ
Kuo et al. [73]	−	+	NR	NA	+	NA	+	+	−	+	+	NR	+	−	MQ
Langenskiöld et al. [76]	+	+	NR	NA	NR	+	+	+	−	+	+	NR	+	−	MQ
Lim et al. [75]	+	+	NR	NA	NR	+	+	+	+	+	+	NR	+	+	HQ
Lind et al. [71]	+	+	NR	NA	NR	+	+	+	+	+	+	−	+	+	HQ
Lind et al. [78]	+	+	NR	NA	NR	+	+	+	−	+	+	−	+	−	MQ
Lind et al. [77]	+	+	+	NA	NR	+	+	+	+	+	+	−	−	+	HQ
Owlia et al. [70]	+	+	NR	+	NR	+	+	+	−	+	+	NR	+	−	MQ
Ribeiro et al. [54]	+	+	NR	−	NR	+	+	+	−	+	−	−	−	−	LQ
Ribeiro et al. [72]	+	+	NR	+	+	+	+	+	+	+	+	+	+	+	HQ
Thanathornwong et al. [88]	−	+	NR	NA	NR	+	NR	+	−	−	+	NR	+	−	LQ

Notes: Abbreviations: + met criteria; − did not meet criteria; NR, not reported; NA, not applicable; HQ, high quality; MQ, moderate quality; LQ, low quality. Questions: 1. Research question/objective clearly stated; 2. Study population clearly specified and defined; 3. Participation rate of eligible persons ≥50%; 4. Subjects recruited from same/similar populations; 5. Sample size justification; 6. Exposure(s) measured prior to outcome(s); 7. Sufficient timeframe; 8. Dependent variable measured in category or as continuous variable; 9. Independent variables clearly defined and measured appropriately; 10. Dependent variables assessed more than once; 11. Dependent variable clearly defined and adequately assessed; 12. Blinding of assessors; 13. Loss to follow-up after baseline of ≤20%; 14. Adjusted for key confounding variables.

**Table 5:** Methodological quality assessment based on the NHLBI controlled intervention studies tool.

Study	Criteria													Rating	
	1	2	3	4	5	6	7	8	9	10	11	12	13	14	
Ribeiro et al. [54]	+	−	−	−	NR	−	−	+	NR	NR	−	−	NR	+	LQ
Ribeiro et al. [72]	+	+	+	+	+	+	+	+	NR	NR	+	+	+	+	HQ

Notes: Abbreviations: + met criteria; − did not meet criteria; NR, not reported; HQ, high quality; LQ, low quality. Questions: 1. Study description, randomized RCT; 2. Adequate method of randomization; 3. Concealed treatment allocation; 4. Providers and participants blinded; 5. Assessors blinded the participants; 6. Baseline characteristics that could affect outcomes; 7. Endpoint dropout rate of ≤20%; 8. Endpoint dropout rate between treatment groups of ≤15%; 9. High adherence to intervention protocols in each group; 10. Other interventions avoided or similar in the group; 11. Outcomes assessed using valid and reliable measures; 12. Sample size justification; 13. Prespecified analysis of outcomes reported; 14. Randomized participants analyzed in original group.

### 3.2. Study Design, Methodology, and Instruments

#### 3.2.1. Study Design

As shown in Table 6, ten of the studies employed a cross-sectional design, while three utilized a combination of cross-sectional and semi-longitudinal or longitudinal prospective approaches. Additionally, one study employed a cluster-randomized controlled trial design. Five studies included a control group, while the rest evaluated the feedback’s effects against baseline measurements or employed a cross-over design.

#### 3.2.2. Work Setting, Work Tasks, and Participants

As shown in Table 7, four studies were conducted in real work settings, involving actual productive tasks, while the remaining ten were carried out in laboratory environments or training facilities. The most common activity was manual handling (n = 10, including one instance of light manual handling). Additionally, five studies focused on care tasks such as patient transfer, two studies examined computer work, and two explored warehouse logistics tasks.

In total, 520 participants were included (median: 16), with 311 participants receiving feedback (median: 13). In four of the fourteen studies, the majority (i.e., >60% of the participants) were women, in four the majority were men, and in five a balanced sample (i.e., 40–60% of each sex) of women and men were included. The gender distribution was not reported in one study [86]. Regarding age, eight studies involved participants aged 20–29 years, while four studies involved participants aged 30–39 years, and two studies involved participants aged 40–49 years. In the five studies conducted in real work settings, participants were trained workers performing their regular work tasks, with one study [71] additionally including trained workers performing tasks in a training facility at their workplace. The remaining studies involved novice participants, individuals undergoing formal training, or subjects performing constructed tasks (e.g., computer entry tasks) that somewhat resembled regular tasks, such as those of students.

#### 3.2.3. Feedback

The most common feedback modalities were vibration (n = 9), auditory (n = 7), and visual (n = 1). In most studies, only one feedback modality was evaluated (n = 12), while one study provided either vibration or audio feedback (based on the preference of the participants) [85], and one study combined all three modalities [87]. All studies evaluated corrective feedback (also referred to as negative feedback [45]), whereas both corrective feedback and reinforcing feedback (also referred to as positive feedback [45]) were evaluated by Langenskiold et al. [76]. Eleven studies evaluated concurrent feedback, where the feedback is triggered when an angular or postural threshold is exceeded. Five of these studies used cumulative concurrent feedback, where the feedback is triggered when the postural threshold is exceeded for a predetermined period (e.g., >30 s), and one used fading feedback, where the feedback is not provided each time the criteria are reached. In one study, terminal feedback was evaluated, in which the feedback is provided after a fixed interval when certain criteria are fulfilled. Notably, the feedback in all studies was triggered by the system (system-determined) and not by the user. As shown in Table 8, the evaluation of feedback effects was most frequently conducted during the feedback delivery phase (n = 10) and immediately after (n = 8). Notably, only one study assessed the long-term effects of feedback, examining outcomes after 12 months or more following feedback administration [72].

As shown in Table 9, the back (including the lower back and trunk), was the most commonly targeted body region (n = 12), followed by the arm (n = 3), and the neck (including inclination of the head, n = 2). In twelve studies, the feedback was triggered by one joint or body part, with half of those studies employing one trigger level (n = 6), or two trigger levels (n = 6). For instance, Lind et al. [71,78] provided one intensity at ≥30° of arm elevation (lower intensity of vibration) and another (higher intensity) at ≥60° of arm elevation. Only two studies provided feedback at more than one joint or body part (i.e., the trunk and arm).

In the study by Langenskiöld et al. [76], vibration feedback was provided at both the trunk and one arm, with one intensity level per body part. Similarly, Lind et al. [77] provided vibration feedback at both the trunk and one arm, but with two intensity levels per body part. Another example of combination is that six studies used a combination of postural angle and time period to activate the feedback. For instance, in the study by Ribeiro et al. [72], feedback was triggered when the trunk was flexed forward (lumbopelvic forward bending) for more than 5 s continuously. In the study by Langenskiöld et al. [76], feedback was triggered using a combination of postural threshold (e.g., >30° trunk inclination) with a threshold for the proportion of time (i.e., >10% of the time). The duration of the feedback varied considerably, ranging from a couple of minutes (e.g., [75,76]), to several hours distributed over several weeks [72,85].

#### 3.2.4. Feedback and Motion Capture System

As shown in Table 10, in three studies, commercially available integrated systems were used, capable of measuring, analyzing exposure, and providing feedback. In the other eleven studies, a combination of commercial devices (typically motion capture sensors and hardware like smartphones or laptops) was utilized, often combined with custom-built parts (e.g., software applications for analyzing data and triggering feedback). In terms of posture/motion capture devices, IMUs were the most commonly used (n = 9 studies), followed by accelerometers (n = 4 studies). One study did not report the motion capture sensor type used.

### 3.3. Effectiveness of Feedback in Controlled Environments

The results of the studies evaluating augmented feedback in controlled environments, classified as high- and moderate-quality, respectively, are presented in Table 11 and Table 12. Additionally, a summary of the evidence is provided in Table 13. 

#### 3.3.1. Effect during Feedback Administration

In terms of the high-quality studies, relative reductions in exposure of between 10 and 50% were reported in the four studies, but there were also statistically insignificant reductions. In a few exceptions, there were statistically insignificant increases in exposure in the studies by Lim et al. [75] and Lind et al. [71]. For the moderate-quality studies, reductions in exposure were of similar degrees, and there were also a few statistically insignificant increases in exposures in one study [74]. When summarizing the current evidence of the effectiveness of reducing postural exposure during feedback administration, there were consistent findings among three high-quality studies and four moderate-quality studies that augmented feedback from WSS can reduce postural exposure during feedback administration. Based on this, the evidence that augmented feedback can reduce postural exposure during feedback administration is assessed as strong. 

#### 3.3.2. Effect Directly after Feedback Administration

For the effectiveness directly after (≤4 h), there were consistent findings among two high-quality studies and two moderate-quality studies that augmented feedback from WSS can reduce postural exposure. For two moderate-quality studies there was a mixture of statistically significant effects of reduced exposure and non-significant effects in both directions. Based on this, the evidence that augmented feedback can reduce postural exposure directly after administration is assessed as moderate–strong.

#### 3.3.3. Retained Effects: Short and Midterm

The effectiveness in the short term and midterm was only evaluated in a single study, which was of high quality. The study showed a consistent statistically significant reduction in posture exposure for the tasks that were included in the feedback training but a non-statistically significant reduction in posture exposure for a new task, evaluating the skill transfer. Based on this, there is limited evidence that augmented feedback can reduce postural exposure in the short and midterm for the tasks that were included in the feedback training, and no evidence for its ability to reduce postural exposure in more complex tasks not previously included in the feedback training. 

### 3.4. Effectiveness of Feedback in Real Work Environments

The results of the studies evaluating augmented feedback in real work environments, classified as high- and moderate-quality, respectively, are presented in Table 14 and Table 15. Additionally, a summary of the evidence is provided in Table 16.

#### 3.4.1. Effect during Feedback Administration

In terms of the effectiveness of augmented feedback to reduce postural exposure during feedback administration, there were consistent findings among one high-quality study and one moderate-quality study that augmented feedback can reduce postural exposure while no statistically significant differences were observed by the high-quality RCT study by Ribeiro et al. [72]. Based on this, the evidence that augmented feedback applied in real work environments can reduce postural exposure during feedback administration is considered inconsistent.

#### 3.4.2. Effect Directly after Feedback Administration

For the effectiveness of augmented feedback to reduce postural exposure directly after it has been administered, one high-quality study observed consistent findings for its effectiveness while a study of moderate quality reported a tendency of reduced exposure following feedback being administered, although not statistically significant.

Based on this, the evidence that augmented feedback applied in real work environments can reduce postural exposure directly after it has been administered is considered to be very limited.

#### 3.4.3. Retained Effects: Midterm

The effectiveness after midterm time elapse was evaluated in one moderate-quality study showing consistent findings if reduced exposure, while the high-quality RCT study did not observe any statistically significant effects.

Based on this, the evidence that augmented feedback applied in real work environments can induce retained reduction in postural exposure up to 3 months after feedback administration is considered inconsistent. 

#### 3.4.4. Retained Effects: Very Short, Short, Long, and Very Long Term

The effectiveness of augmented feedback to reduce postural exposure after one week, one month, and up to 12 months was evaluated in two studies, and one study evaluated the effectiveness after 12 months. When considering the results of each of these, there were insignificant results. Based on this, the assessment is that there is currently no evidence that augmented feedback applied in real work environments can induce retained reduction in postural exposures after one week, one month, or 12 months.

## 4. Discussion

### 4.1. General Summary of the Findings

This rapid review identified 16 studies from peer-reviewed journal articles that evaluated the effectiveness of augmented feedback from wearable motion capture systems in reducing upper body postural exposure. Six of the studies were of high quality and eight of moderate quality. The reporting of participation rates, blinding of assessors, and a-priori power calculations were infrequently performed. Four studies were conducted in real work environments, while ten were conducted in controlled environments. Vibration feedback was the most common type utilized (n = 9), followed by auditory feedback (n = 7) and visual feedback (n = 1). All studies employed corrective feedback initiated by the system. Three studies used commercially available systems capable of measuring, analyzing exposure, and providing feedback, while the other 11 studies utilized a combination of commercial devices (typically motion capture sensors and hardware like smartphones or laptops), often combined with custom-built parts. IMUs were the most commonly used motion capture device to initiate the feedback (n = 9 studies), while triaxial accelerometers were used in four studies. 

There was significant heterogeneity across the studies in terms of the type of task evaluated, experience and age of the participants, feedback characteristics (e.g., trigger criteria and modality), training period, follow-up period, and evaluation criteria (e.g., postural cut-offs and ranges), restricting meta-analysis.

In controlled environments, the assessment indicates strong evidence for the effect of feedback during its administration and moderate to strong evidence directly after administration. There is limited evidence for its effectiveness in the short term and midterm for tasks included in the feedback training.

In real work environments, the assessment indicates inconsistent evidence for the effectiveness of feedback in reducing postural exposure during its administration and for retained reduction in postural exposure up to three months after the feedback was last administered. Its effectiveness directly after administration was assessed as very limited. Furthermore, the assessment indicates that there is no evidence that augmented feedback in real work environments can induce sustained reductions in postural exposure after one week, one month, or up to 12 months after the feedback was last administered.

### 4.2. General Interpretation of the Results

When comparing to the systematic review by Frasie et al. [67], who concluded that there was moderate evidence that feedback applied in a controlled environment can improve (what they referred to as) sensorimotor control, there was conflicting evidence when the feedback was applied in the workplace. Frasie et al. [67] had broader inclusion criteria not restricted to only augmented feedback from wearable systems nor from motion sensors, leading to larger heterogeneity in the feedback types evaluated. In contrast, the current review specifically focused on augmented feedback from wearable motion capture systems, resulting in a narrower selection of studies. Notably, the current review identified several high- and moderate-quality studies, such as those by Doss et al. [74], Bootsman et al. [87], Lind et al. [71,77], Lim et al. [75], and Langenskiöld et al. [76] that were not included in Frasie et al.’s review. This expanded inclusion may, at least partly, account for the observed increase in the strength of evidence from moderate to strong in controlled environments during feedback administration.

The review by Lee et al. [66] concluded that, at the time of their review, there was limited evidence supporting the effectiveness of augmented feedback from inertial sensor systems in improving posture and reducing adverse postures. Their assessment did not differentiate between studies conducted in controlled environments and those in real work environments, nor did it consider temporal aspects such as the separation of effects during feedback administration and retention. It may pose a challenge for results coming from these contexts, since tasks performed in controlled environments are typically less complex and exhibit lower exposure variability compared to tasks in real work environments [90]. Examples include productivity demands and incentive structures, elements of the physical environment, and various psychosocial factors, such as perceived time constraints. Additionally, several studies included in Lee et al.’s review, such as those by Breen et al. [91]; Kuo et al. [92]; Vignais et al. [93]; Thanathornwong et al. [94,95]; Brakenridge et al. [96]; Park et al. [97]; and Cerqueira et al. [98], did not meet the inclusion criteria based on methodological quality. Furthermore, since Lee et al.’s review, several high- and moderate-quality studies meeting the inclusion criteria of the current review have been published, including those by Kamachi et al. [6]; Langenskiöld et al. [76]; Lim et al. [75]; and Lind et al. [77]. Additionally, several more studies published before July 15, 2021 were identified, such as those by Bazazan et al. [85]; Lind et al. [71]; Kuo et al. [73]; Owlia et al. [70]; and Ribeiro et al. [72]. The differing conclusions between the current review and Lee et al.’s review may largely be attributed to the increasing number of high- and moderate-quality studies published from 2020 to 2023. 

The strength of evidence in the current review was based on 14 data pools, based on seven temporal categories (ranging from during feedback administration to twelve months or more after feedback administration) and two distinct environments (controlled versus real work). Consistent with Frasie et al. [67], it was deemed appropriate to differentiate between the effects of feedback applied in controlled environments versus real work environments, where the latter typically involves more diverse tasks, as well as other elements that could be relevant to consider when evaluating its effectiveness in the workplace. Hence, the effects observed in controlled environments may not directly translate to more complex work environments. Consequently, it was deemed necessary to evaluate the effects separately for studies conducted in controlled environments and those conducted in real work environments. The efficacy of transferring training to reduce exposure to tasks not explicitly covered in the training remains uncertain.

Kamachi et al. [79] evaluated this aspect and found that while the feedback training group did not significantly reduce their exposure in the unfamiliar task compared to the control group, they did so for the caregiving task included in the feedback training. An alternative hypothesis suggests that the effectiveness of using augmented feedback to reduce the occurrence of adverse postures depends largely on the work context, particularly the ability to adjust work postures and movements beneficially during tasks. Thus, improved work techniques may have limited potential to reduce exposure in certain situations, necessitating other measures to achieve acceptable exposure levels. As highlighted by Lind et al. [45], augmented feedback should be viewed as a complementary approach alongside other strategies, and should typically adhere to the hierarchy of controls [22].

While Frasie et al. [67] analyzed the evidence using seven temporal categories (during; just after the intervention; <2 months; <6 months; and ≥6 months), it was deemed appropriate to incorporate more categories to enhance differentiation between shorter- and longer-term effects. Having an extended number of categories could potentially result in lower evidence as there are fewer studies per group, but it could also strengthen the grading of the evidence by having fewer conflicting results. For instance, there was inconsistent evidence in the midterm when feedback was applied in a real work environment, and no evidence for an effect in the very short and short term, which seems counterintuitive assuming that the effect feedback gradually fades, as indicated in several studies (e.g., Kamachi et al. [6] and Lind et al. [77]).

Additionally, is worth noting that the literature is relatively scarce regarding the effectiveness of feedback from wearable motion capture systems in both controlled environments and real work environments for follow-up periods of more than 4 h. The one exception was studies performed in controlled environments evaluating the effect during feedback administration and immediately after its application (i.e., ≤4 h), for which there were seven and five studies, respectively. Of the 16 studies that met the inclusion criteria and were assessed for methodological quality, the oldest study was published in 2014, while the oldest study included after methodological quality assessment was published in 2019. Therefore, the grading of the evidence should be considered within the context of a rapidly evolving research field, and the assessment of evidence may shift with the inclusion of new studies of moderate and high quality.

No meta-analysis was undertaken due to the notable heterogeneity among the studies, which encompassed variations in, e.g., study designs, methodologies, participant demographics, and outcome measures. Consequently, a descriptive synthesis was employed to systematically summarize the findings and used as base for grading the evidence.

#### 4.2.1. Application of Systems and Sensors

Vibration and auditory feedback emerged as the most common modalities in the current review, aligning with findings from Figueira et al. [69]. Conversely, García-Jaén et al. [68] noted auditory feedback as the most common modality, while visual and/or vibrotactile feedback was most common in the review by Lee et al. [66]. These discrepancies could be attributed, at least in part, to variations in scope and inclusion criteria. For instance, the current review specifically focused on wearable systems, thereby excluding studies utilizing stationary visual displays.

All studies, with the exception of Bazazan et al. [85], reported using accelerometers or IMUs to monitor postural exposure and using this information to initiate feedback. Notably, none of the feedback systems employed motion parameters, such as velocity or acceleration, to trigger the feedback, although trunk kinematics was evaluated as an outcome by Doss et al. [74]. Instead, feedback was initiated based on postural thresholds or cumulative time spent above a certain posture threshold. Incorporating motion as a trigger for feedback may be pertinent in future research, given its association with MSDs [99,100,101,102,103,104,105] and may be possible to utilize, at least for the systems that comprise IMUs, as using accelerometers alone can lead to significant overestimations of angular velocity [106,107,108].

Among the twelve studies identified by Figueira et al. [69], the majority (n = 7) utilized accelerometers to provide feedback, while five utilized IMUs. Conversely, in the current review, IMUs were most commonly used (nine studies), whereas accelerometers were used in four studies. Most of the systems in the current review were custom-built, with the so-called Smart Workwear System (including the ErgoRiskLogger smartphone application) utilized in four studies and the PostureCoach in three studies. It should be noted that different versions and configurations of the custom system were used across the studies.

#### 4.2.2. Effectiveness of Different Types of Feedback

Interestingly, all studies evaluated corrective feedback whereas Langenskiold et al. [76] also assessed reinforcing feedback in one group alongside corrective feedback in another. However, due to the small number of participants in each group (i.e., five versus four), this analysis was not included because it did not meet the minimum sample size required for statistical power according to the inclusion criteria. Consequently, it is not possible based on the current review to determine whether corrective or reinforcing feedback is more effective. Some previous research suggests that corrective feedback is effective in facilitating motor learning [109,110], particularly among skilled individuals [109,111]. However, concurrent correction feedback may, on the other hand, inhibit the body’s intrinsic feedback system as well as induce dependence on external feedback if administered often or for prolonged durations [61,112,113]. Hence, more studies are needed that compare different types of feedback. The same applies for the feedback modality, in which vibration and auditory feedback were the most commonly used. The effectiveness of one feedback modality over another likely depends on its application [109]. For instance, visual feedback can be less suitable for tasks requiring continuous visual attention and auditory feedback where there is considerable background noise, or where it can disturb surrounding individuals [85]. Combining several feedback modalities simultaneously has been suggested to improve feedback effectiveness [109]. However, all but two studies in the current review provided only one type of feedback modality. A remark to this is that vibration feedback commonly produces unintended noise from the vibration motor that sometimes is amplified via connecting materials [78]. This side effect could potentially enhance the effectiveness of vibration feedback, making it challenging to attribute the effect solely to its haptic components, especially in environments with low background noise. Consequently, vibration feedback may need to be classified and evaluated as a combination of both vibration and auditory feedback in some applications.

#### 4.2.3. Study Samples

In terms of the gender distribution across the studies, an equal number had a majority of women as those with a majority of men, while five studies included a balanced proportion of both genders. However, regarding age distribution, most participants in the studies were younger adults aged under 30 years, with none of the studies having a mean age exceeding 50 years. Therefore, there may be a need for greater diversity in participant age to better reflect the age distribution in the working population. Moreover, the majority of studies focused on untrained subjects. Given the potential variation in the effectiveness of augmented feedback between trained and untrained individuals, there is an opportunity for future research to investigate this aspect further. Specifically, future studies could assess the effectiveness of augmented feedback while considering participants’ prior training and could further include a greater proportion of trained participants familiarized with the tasks evaluated.

As previously noted, only four out of the initial 16 studies eligible for methodological quality assessment provided satisfactory justification for their sample size through a priori statistical power calculation. Among those that did not report such justification, sample sizes appeared to be based on previous studies, as observed in studies like Owlia et al. [70] Thanathornwong et al. [88] and Lind et al. [77]. Additionally, considering the indication that the effectiveness of feedback training may gradually decrease over time, future studies should adjust their sample size calculations to account for this phenomenon, also considering potential dropout rates corresponding to the study duration.

### 4.3. Limitations

#### 4.3.1. Limitations of the Evidence

There are several limitations to consider when interpreting the results of this rapid review. Firstly, the evidence of the effectiveness of augmented feedback in reducing postural exposure only pertains to studies utilizing wearable systems and motion capture systems targeting the upper body. Consequently, the effectiveness of augmented feedback in reducing postural exposure when considering non-wearable systems or other types of exposure input, such as sEMG, may differ. Additionally, the evidence for its effectiveness may vary for different body regions, such as the lower limbs. 

Furthermore, the review did not assess the potential effectiveness of augmented feedback for rehabilitation purposes, such as for individuals with specific medical conditions, nor did it consider populations younger or older than the general working population. Moreover, the findings from the review may not directly translate to the effectiveness of preventing WMSDs, nor does it account for the possibility that work technique training targeting single body parts may transfer exposure to other body parts. With regards to the latter, most of the studies targeted a single body part or joint, potentially leading users to adopt compensatory strategies that decrease exposure in the targeted area but may transfer or increase exposure in other body parts, similar to what has been observed in some studies evaluating the effects of occupational exoskeletons [114]. In Lind et al. [71] for example, visual observations of the participants indicated that the participants sometimes bent their knees and hence moved the upper body vertically, which likely increases the metabolic load. Additionally, there is also a possibility that biofeedback introduces other unintended adverse effects, for example, observed reduced performance as a result of biofeedback, indicating increased cognitive load [115].

While the literature review offers limited insight into the longer-term effects of augmented feedback, extending beyond several weeks and months, it is generally challenging to isolate the effect of a single intervention in real work environments. As discussed by Winkel and Westgaard [116], work environments undergo continuous changes to enhance the production system performance. These changes can influence the parameters studied in the intervention and may attenuate the reduction in exposure attributed to the intervention [117,118].

The heterogeneity of tasks included can be perceived as both a strength and a weakness. While this diversity makes it challenging to compare the effectiveness of various feedback modalities and timing, it offers the advantage of evaluation across different types of tasks, as opposed to a limited focus on one or two specific tasks. However, it is essential to exercise caution when generalizing the effectiveness or ineffectiveness of feedback to significantly different tasks or contexts than those covered by the included studies.

The grading of evidence did not consider the possibility that certain feedback strategies, such as training set-up or duration, may be more effective than others. Thus, it may be advantageous to categorize studies based on additional criteria. This contrasts with previous discussions on ergonomics interventions, where the grading system for evidence may prioritize study quality over intervention quality, highlighting potential limitations in the current approach [119].

Most of the systems utilized in the studies were custom designed for research purposes, primarily aimed at evaluating the effectiveness of augmented feedback rather than as intervention projects. Consequently, there may be additional barriers to their implementation, including issues related to usability, such as ease-of-use [7,32], which have been identified as hindrances for the application of wearable technology [120] as well as the durability of the technology [121], among others.

#### 4.3.2. Limitations of the Review Processes

This review adheres to the guidelines for rapid reviews [80], which entails a less rigorous process compared to systematic literature reviews following the PRISMA guidelines. Therefore, the limitations commonly associated with rapid reviews also apply here. However, given the rapid advancements in the field, rapid reviews offer the advantage of a quicker process from literature search to the publication of results. Consequently, this can lead to a more up-to-date review that includes recently published studies. There were some deviations from the Cochrane rapid review methods recommendations that could be highlighted. As recommended, (at least) three databases were used as primary sources to identify literature, but the full screening of all of the titles and abstracts was performed by one researcher, instead of two researchers screening 20% of the titles and abstracts. As per the Cochrane recommendations, one reviewer screened all included full-text articles, but contrary to the recommendations no second reviewer screened the excluded full-text articles. Despite these deviations, the literature search identified several studies that were not included in previous reviews but appeared to meet their inclusion criteria. 

In accordance with Cochrane recommendations for rapid reviews, one reviewer assessed the methodological quality, but this assessment was not verified by a second reviewer. Furthermore, the cut-off for determining moderate and high quality was more conservative compared to some previous reviews, which used cut-offs of 33% and 66%, respectively, for moderate and high quality [83,122]. 

Cut-offs for determining methodological quality are somewhat arbitrary, and there is no general consensus on which cut-offs are most suitable [123]. In this review, the cut-offs were based on the assessment of 14 criteria. It was judged that studies fulfilling less than half of the criteria could induce a non-negligible risk of bias. Therefore, a sum score of ≥75% was used as the cut-off for high-quality studies, and ≥50% for moderate-quality studies.

Contrary to Lee at al. [66], who included conference papers, the inclusion criteria were set to exclude sources other than peer-reviewed scientific journal articles. This was done to ensure that all included studies had undergone a rigorous peer-review process involving at least two independent reviewers. This exclusion criterion may have resulted in the omission of some otherwise relevant studies, which could have potentially influenced the grading of the evidence (e.g., [91,94,124,125,126,127,128]). The same consideration applies to studies with fewer than eight subjects receiving feedback without an a-priori based in power calculations justifying the smaller sample size (e.g., [91,93,98,124,129,130,131]). While the eight-subject cut-off may seem somewhat arbitrary, it was chosen to minimize the likelihood of both type I and type II errors.

### 4.4. Practical Implications and Future Research

Several measures can enhance the methodological quality of future studies on the effectiveness of augmented feedback. A few of the included studies reported crucial details such as the participation rate of eligible individuals, blinding of assessors, and justification for statistical power. The diverse range of studies examined in this review can aid in facilitating power calculations for future research, particularly if no pilot study has been conducted to estimate effect sizes, although a pilot study is typically necessary given the diverse magnitude of the effect of the feedback (including no significant effects).

Another critical consideration is the need to control or measure the work rate during interventions. Failure to do so could result in the observed reduction in exposure being attributed primarily to a lower work rate rather than the intervention itself. Analogously to this is the issue of intervention studies aimed at increasing exposure variability or reducing MSD symptoms using work or task rotation. In some of these studies, the maintenance of a constant work rate was not ensured, potentially resulting in changes in exposure being largely attributed to breaks or a reduced amount of work performed [132]. The importance of controlling the work rate may vary depending on the study design. While it may be less critical for randomized controlled trials (RCTs) collecting data over an extended period, it becomes more relevant for RCTs with a cluster design or studies with a limited data collection timeframe, such as those spanning only a few hours or days.

Several research gaps have been identified, including the effectiveness of augmented feedback training attributed to individual factors and contextual factors, as well as feedback characteristics and training programs. Partially unexplored individual factors include the effectiveness of feedback based on the experience level of recipients (i.e., novice versus experienced participants performing their regular work) and potential differences associated with age (i.e., older versus younger participants). Insufficiently explored feedback characteristics include whether one type of feedback modality is more effective compared to others and in which contexts, and whether feedback applied to multiple locations, such as both the trunk and arm, influences effectiveness and retention. Future research should focus on evaluating the effectiveness of different feedback characteristics within studies (e.g., corrective versus reinforcing feedback, or vibration versus auditory feedback) due to the heterogeneity between studies, including their specific contexts and potential to reduce exposures. Related to this is the need to systematically identify contexts where feedback has the greatest potential to reduce postural exposure and to design effective feedback training programs that maximize long-term retention effects. Additionally, future studies should evaluate potential unintended adverse effects of augmented feedback, such as its influence on cognitive load and the transfer of exposure to other body parts. Lastly, more research is needed to evaluate the longer-term effects of feedback in both controlled and real work environments due to the relatively few longer-term studies currently available.

While there is moderate to strong evidence supporting the effectiveness of augmented feedback from wearable motion capture systems in reducing postural exposure during and immediately after feedback administration in controlled environments, its longer-term effects remain largely unexplored. Therefore, based on the current evidence, there is insufficient evidence for a general recommendation for the use of augmented feedback from wearable motion capture systems as a risk-reducing and preventive measure for WMSD. Consequently, further research is warranted to assess its potential as a risk-reducing measure for reducing postural exposure and work-related musculoskeletal disorders (WMSDs).

## 5. Conclusions

This rapid review assessed the use and the current evidence for the effectiveness of work technique training utilizing augmented feedback through wearable motion capture systems to reduce postural exposure. Six studies of high quality and eight studies of moderate quality met the inclusion criteria and were summarized descriptively, and the strength of evidence for the effectiveness of augmented feedback was assessed. Four studies were conducted in real work environments, while ten were conducted in controlled settings. Vibration feedback was the most common feedback type utilized (n = 9), followed by auditory (n = 7) and visual feedback (n = 1), all employing corrective feedback initiated by the system. 

In controlled environments, the assessment of the current evidence based on high- and moderate-quality studies indicates strong evidence for the effect of feedback during its administration and moderate to strong evidence for its effect directly after administration. However, there is limited evidence for its effectiveness in the short term and midterm for tasks included in the feedback training, and no evidence for its ability to reduce postural exposure in more complex tasks not previously included in the training. 

In real work environments, the assessment of the current evidence based on high- and moderate-quality studies indicates inconsistent evidence for the effectiveness of feedback in reducing postural exposure during its administration and for retained reduction in postural exposure up to three months after the feedback was last administered. Its effectiveness directly after administration is considered to be very limited, and the compiled available data indicates that there is no evidence that augmented feedback in real work environments can induce sustained reductions in postural exposure after one week, one month, or up to 12 months after the feedback was last administered.

The current literature on the effectiveness of augmented feedback from wearable motion capture systems in both controlled and real work environments, especially for follow-up periods exceeding 4 h, is relatively scarce. Consequently, the grading of the evidence, especially for follow-up periods exceeding 4 h, may be subject to change in light of new studies of moderate and high quality. Therefore, further research is needed, particularly research with a longitudinal design and conducted in real work environments. The review identified several actions to enhance the methodological quality of future studies, such as reporting the participation rate of eligible individuals and ensuring sample sizes are supported by a priori calculations of statistical power.

## Figures and Tables

**Table 1:** The eligibility criteria for including and excluding studies.

Eligibility Criteria	Descriptions
Evaluating wearable instruments or systems that monitor postures or movements of the upper body (i.e., neck/head, trunk, arms, or wrist/hand) and provide feedback to the user (wearer) based on this information.	Instruments or systems that are not ambulatory, such as those that are depending on fixed instruments (e.g., video-based motion tracking systems) are not included. Additionally, instruments or systems that do not directly base the feedback on postures and/or movement of the upper body (e.g., muscle activation using sEMG) are not included. Only instruments or systems providing feedback directly to the wearer were considered (i.e., not via an instructor). Studies using other types of tools to provide feedback, such as elastic bands or dowels, are also excluded. Evaluations of activities targeting the lower part of the body, such as legs or feet (e.g., studies focusing on gait), are also excluded.
Have evaluated feedback on real work tasks or tasks closely resembling real work tasks, and reported the effects of postures and/or movements of the upper body.	Studies involving activities with a low resemblance to work-related tasks or where work-related tasks are not reported separately from leisure time activities are excluded. Examples include tasks where participants are instructed to move their arms to follow a pre-set trajectory in space or manipulate non-physical (virtual) objects. Similarly, studies where participants are instructed to sit on unusual objects without performing work-related tasks are also excluded. However, tasks that closely mimic real work, such as computer typing (even if not an actual paid task), are considered to meet the inclusion criteria. Furthermore, studies solely focusing on outcomes such as usability and wearability are not included.
Having an adult population aged 18–67 years who are not from a specific patient population.	Studies involving subjects from specific patient populations, such as those with particular medical conditions, are excluded. However, studies including a normal working population, where musculoskeletal disorders (MSDs) could occur, but participants were not restricted from performing their work tasks, are deemed to meet the inclusion criteria.
Having at least eight participants receiving the feedback, and the effect of feedback is tested statistically.	A sample size lower than eight participants is acceptable only if it has been justified based on power calculations. This implies that in the final data analysis, there should be a minimum of eight participants aged 18–67 receiving feedback, or at least eight participants for each type of feedback included (in cases where more than one feedback type is provided).

**Table 2:** The criteria for assessing the duration categories (feedback follow-up duration).

Duration Classification	Criterion/Criteria (Time Elapsed)
During feedback	Simultaneous to feedback administration
Directly after	Directly after, up to ≤4 h after feedback administration
Very short term	More than four hours, and up to ≤1 week after feedback administration
Short term	More than one week, and up to ≤1 month after feedback administration
Midterm	More than one month, and up to ≤3 months after feedback administration
Long term	More than 3 months, and up to <12 months after feedback administration
Very long term	Twelve months or more after feedback administration

**Table 3:** The criteria for assessing the strength of evidence.

Strength of Evidence	Criteria
Strong evidence	Consistent findings among three or more studies of at least medium quality, including a minimum of two high-quality studies.
Moderate evidence	Consistent findings among two or more studies of at least medium quality, including at least one high-quality study.
Limited evidence	Findings from at least one high-quality study or two moderate-quality studies.
Very limited evidence	Findings from one moderate-quality study.
Inconsistent evidence	Inconsistent findings among multiple studies (e.g., one or multiple studies reported a significant result, whereas one or multiple studies reported no significant result).
Conflicting evidence	Contradictory results between studies (e.g., one or multiple studies reported a significant result in one direction, whereas one or multiple studies reported a significant result in the other direction).
No evidence	Insignificant results derived from multiple high or medium quality studies.

**Table 6:** Summary of study objective and design, and comparison group.

Study	Objective	Study Design	Comparison Group
Ailneni et al. [84]	Evaluating the effectiveness of vibration feedback in reducing flexion/inclination angles of the head and neck, as well as gravitational moment on the neck during sitting and standing computer work.	CS	no CG
Bazazan et al. [85]	Evaluating the effectiveness of augmented feedback in preventing slouching or postural kyphosis and occurrence of musculoskeletal symptoms and fatigue among control room operators.	CS/LN	CG
Boocock et al. [86]	Evaluating the effectiveness of real-time external biofeedback to modify lumbosacral posture and trunk flexion in repetitive lifting task compared to no biofeedback.	CS	CG
Bootsman et al. [87]	Evaluating the effectiveness of augmented feedback in reducing episodes of lower back flexion	CS	no CG
Doss et al. [74]	Evaluating the effectiveness of augmented feedback on reducing peak trunk kinematics, e.g., flexion angle, velocity, and acceleration, in patient transfer tasks.	CS	no CG
Kamachi et al. [79]	Evaluating the effectiveness of augmented feedback to reduced time in end-range lumbar spine flexion while performing care tasks, and skill transfer to other tasks.	CS/SLN	CG
Kuo et al. [73]	Evaluating the effectiveness of vibration feedback to reduce occurrence of slouched postures in seated computer task	CS	no CG
Langenskiöld et al. [76]	Evaluating the effectiveness of vibration feedback to reduce time in adverse trunk inclination angles and dominant upper arm elevation angles	CS	no CG
Lim et al. [75]	Evaluating the effectiveness of vibration feedback to reduce sagittal trunk flexion angles in construction work tasks by providing vibrotactile feedback and compare the effectiveness of two feedback locations.	CS	no CG
Lind et al. [71]	Evaluating the effectiveness of vibration feedback to reduce time in adverse trunk inclination angles and arm elevation angles in simulated industrial order picking.	CS	no CG
Lind et al. [78]	Evaluating the effectiveness of vibration feedback to reduce time in arm elevation angles in letter sorting.	CS	no CG
Lind et al. [77]	Evaluating the effectiveness of vibration feedback to reduce time in adverse trunk inclination angles in real warehouse order picking.	CS/SLN	No CG
Owlia et al. [70]	Evaluating the effectiveness of auditory feedback to reduce peak lumbar spine flexion in caregiving tasks.	CS	CG
Ribeiro et al. [72]	Evaluating the effectiveness of a lumbopelvic monitor and extrinsic feedback device to reduce occurrence of trunk inclination.	cluster RCT	CG

Notes: Abbreviations: CS, cross-sectional; SLN, semi-longitudinal; LN, longitudinal; RCT, randomized control trial; CG, control group.

**Table 7:** Summary of study settings and tasks, and participants ‘characteristics.

Study	Setting	Tasks	Participants (Mean (SD): Age, Body Mass, Stature) and Eligibility
Ailneni et al. [84]	Lab	Computer typing	Nineteen participants (ten females and nine males), 24.5 (5.3) years, 66.8 (9.3) kg, 168.0 (12.3) cm. Eligibility: have a typing speed of at least 30 words per minute, have not had any pain in the upper extremities or lower back region within the past 7 days.
Bazazan et al. [85]	Real work	Control room operations	A total of 188 control room operators (all male). Control group: 33.1 (4.0) years; body mass: NR; stature: NR; body mass index 25.5 (3.0). Intervention (feedback) group: 32.8 (5.3) years; body mass: NR; stature: NR; body mass index 25.0 (2.7). Eligibility: being a full-time control room operator with at least 1 year working experience, having no apparent physical and mental problem (self-reported).
Boocock et al. [86]	Lab	Manual lifting and lowering a box	A total of 36 university students ^1^ (sex not reported) that were not experienced in manual handling or performed regular handling in their work. Control group (n = 16): 25.6 (5.1) years, 85.5 (13.8) kg, 1.84 (0.08) m. Intervention (feedback) group (n = 18): 25.7 (4.6) years, 79.8 (11.2) kg, 1.80 (0.08) m. Eligibility: no back injury or complaint in the last six months, not having undergone spinal surgery, not having a cardiovascular or neurological condition, and not having a musculoskeletal injury at the time of the study.
Bootsman et al. [87]	Real work	Intensive care and home care tasks	Thirteen nurses (all female), 39.8 (13.6) years, body mass: NR, stature: NR. Eligibility: Not having a sedentary job and not suffering from low back pain.
Doss et al. [74]	Lab	Patient transfer	Ten nursing students (all female), 26.1 (9.1) years, 61.7 (13.5) kg, 1.7 (0.08) m. Eligibility: no history of back pain in the last 12 months.
Kamachi et al. [79]	Lab ^6^	Patient transfer	Twenty healthy adults with no formal training in caregiving or patient handling (10 female, 10 male). Control group: (five female, five male), 23.6 (3.1) years, 75.6 (16.9) kg, 175.8 (9.2) cm. Intervention group: (five female, five male), 24.4 (3.7) years, 76.8 (8.2) kg, 177.6 (8.2) cm. Eligibility: being able to speak and understand English, not having previous caregiving experience or healthcare provider training, no back pain in the past six months or any musculoskeletal disorders related to the spine, and no musculoskeletal issues related to the spine.
Kuo et al. [73]	Lab	Computer work	A total of 21 healthy young adults from university campus (twelve women, nine men), 23.3 (±2.9) years, 61.4 (±10.0) kg, 167.0 (±9.0) cm. Eligibility: age between 20–25 years.
Langenskiöld et al. [76]	Lab	Office-type of manual handling tasks ^2^	Ten participants ^3^ (eight women, two men, nine administrative office workers, and one industrial manual handler), 43.9 (12.0) years, 74.2 (10.6) kg, 166.6 (9.4) cm. Eligibility: not having restrictions in movement or pain from the dominant shoulder/arm, back, hip or knees.
Lim et al. [75]	Lab	Construction tasks ^5^	Fourteen healthy male participants (fourteen men, zero women), 26.1 (4.6) years, 75.4 (8.6) kg, 175.8 (38.2) cm. Eligibility: participants of 18–35 years with no construction work experience, and that have not received any formal training on safe construction work techniques and without preexistence of MSDs.
Lind et al. [71]	Lab ^7^	Manual warehouse order picking	Fifteen ^4^ warehouse workers (twelve men, three women), 39 (12) years, 88 (22) kg, 181 (10) cm. Eligibility: participants without musculoskeletal discomfort or disorders that could hinder the order picking task were included.
Lind et al. [78]	Lab	Manual mail (letter) sorting to letter trays	Sixteen university students and staff (nine women, seven men), 25 (8) years, 70 (13) kg, 170 (13) cm. Novice (i.e., <3 months’ experience of mail sorting) Eligibility: Not having musculoskeletal discomfort or disorders that could hinder mail sorting.
Lind et al. [77]	Real work	Manual warehouse order picking	Fifteen warehouse order pickers (fourteen men, one woman), 30.8 (11.5) years, 77.1 (11.3) kg, 179.3 (8.1) cm. Eligibility: currently working as a warehouse order picker and not having disorders or pain that would prevent performing regular work.
Owlia et al. [70]	Lab ^6^	Patient transfer	Twenty healthy adults with no formal training in caregiving or patient handling (10 female, 10 male). Control group: (six female, four male), 24.7 (2.7) years, mass (62.8 (10.2) kg, 172.1 (8.3) cm. Intervention group: (four female, six male), 28.1 (6.4) years, 71.3 (16.3) kg, 171.6 (7.2) cm. Eligibility: not reported, but seemed to target adult s (i.e., ≥18 years), participants with no formal training in caregiving or patient handling, that can speak and understand English, and that have no history of back pain in the last six months and no musculoskeletal issues related to the spine.
Ribeiro et al. [72]	Real work	Health care	A total of 130 healthcare workers (110 women, 20 men), 45.3 (13.2) years, 70 (range: 61–84) kg, 162.6 (7.9) cm. Feedback group (53 women, 10 men), 48 (range: 36.5–55.0) years, 68 (range: 60–83.1) kg, 162.2 (7.5) cm. Control group: (57 women, 10 men), 47 (range: 31.5–56.0) years, 75 (range: 62–86) kg, 162.9 (8.4) cm. Eligibility: adult health care workers who were performing their regular work activities without any limitations such as limitations due to LBP or LBP symptoms and who are working at least 20 h per week.

Notes: NR, not reported; ^1^ the data of 31 participants were analyzed, i.e., 16 novice participants in the no-feedback group (100%) and 15 (83%) in the feedback group completed the full trial (hence were included in the analysis); ^2^ tasks: sorting letters and binder, lifting smaller and larger boxes; ^3^ data analyzed for nine participants; ^4^ the data of two participants were excluded from the final analysis due to technical issues; ^5^ tasks: lifting-lowering, shoveling, and tying rebar; ^6^ HomeLab at Toronto Rehabilitation Institute; ^7^ training facility in a real work setting.

**Table 8:** Summary of when the feedback was administered.

Study	Feedback Follow-Up Duration	Description of Design
Ailneni et al. [84]	During feedback	Order (counterbalanced order of the conditions ^1^), no baseline: (a) sitting (30 min ^2^, no feedback), (b) sitting (30 min ^2^, feedback), (c) standing (30 min ^2^, no feedback), (d) standing (30 min ^2^, feedback). Feedback condition duration: about 60 min
Bazazan et al. [85]	Midterm (≤3 months) Long term (≥6 to <12 months)	Order: (a) baseline (no feedback); (b) feedback condition: feedback 30 min two times per workday for 12 weeks; (c) about 3 months after feedback condition (no feedback); (d) about 9 months after feedback condition (no feedback). Feedback condition duration: up to about 60 h
Boocock et al. [86]	During feedback	Order (no baseline): lifting for 20 min (feedback group: with feedback, control group: without feedback). Feedback condition duration: about 20 min
Bootsman et al. [87]	During feedback Directly after (≤4 h)	Order: (a) baseline (30 min, no feedback); (b) feedback condition (60 min, feedback); (c) retention test condition (60 min, no feedback); (d) feedback condition (60 min, feedback ^3^). Feedback condition duration: 120 min (60 + 60)
Doss et al. [74]	Directly after (≤4 h)	Order: (a) baseline: three tasks each four times (<5 min, no feedback); (b) feedback session: three tasks each eight times (<10 min, feedback); (c) post-feedback session: three tasks each four times (<5 min, no feedback). Feedback condition duration: <10 min
Kamachi et al. [79]	Directly after (≤4 h) ^4^ Short term (≤1 month) Midterm (≤3 months)	Order day 1: Trial 1 (~15 min, no feedback: intervention group and control group), video training (intervention group and control group); Trials 2 and 3 (each ~15 min, feedback 100% ^8^: intervention group, no feedback: control group); Trial 4 (~15 min, no feedback: intervention group and control group). Order day 2: Trial 5 (~15 min, no feedback: intervention group and control group); Trials 6 and 7 (each ~15 min, feedback 50% ^8^: intervention group, no feedback: control group); Trial 8 (~15 min, no feedback: intervention group and control group). Follow-up tests after 2 weeks (trial 9) and 2 months: Trials 9 and 10: previous tasks and a new task to test the skill transfer, no feedback for either group. Feedback condition duration: about 60 minutes ^5^
Kuo et al. [73]	During feedback	Order: random assignment to either conditions A or B: Condition A: 1 h without feedback then directly after 1 h with feedback; Condition B: 1 h with feedback then directly after 1 h without feedback. Feedback condition duration: about 1 h
Langenskiöld et al. [76]	During feedback Directly after (≤4 h)	Order: (a) practice session, (b) baseline session (no feedback) 4–6 min, (c) intervention session (feedback) 8–12 min, (d) post-intervention session (no feedback) 4–6 min. Feedback condition duration: 8–12 min
Lim et al. [75]	During feedback	Random order (conditions and tasks): three feedback conditions each performed in three tasks. Tasks: lifting/lowering (3.4 ± 1.5 min), shoveling (7.2 ± 1.3 min), rebar tying (6.9 ± 1.1 min) Feedback conditions: (a) no feedback, (b) feedback from vibration motor on the back, (c) feedback from vibration motor on the wrist Feedback condition duration: about 35 min
Lind et al. [71]	During feedback Directly after (≤4 h)	Order: (a) practice session (no feedback), (b) baseline session (~6 min, no feedback), (c) intervention session 1 (~6 min, feedback), (d) intervention session 2 (~6 min, feedback), (e) post-intervention session (~6 min, no feedback). Feedback condition duration: about 12 min
Lind et al. [78]	During feedback	Order: (a) practice session (no feedback), (b) baseline session (~1 min, no feedback), (c) work design session ^9^ (no feedback), (d) ergonomics instruction session 1 (~1 min, no feedback), (e) intervention session 1 (~1 min, feedback), (f) ergonomics instruction session 2 (~1 min, no feedback), (g) intervention session 2 (~1 min, feedback). Feedback condition duration: about 2 min
Lind et al. [77]	During feedback Directly after (≤4 h) Very short term (≤1 week) Short term (≤1 month)	Order ^6^: (a) baseline session (~45 min, no feedback), (b) feedback session 1 (2 days after baseline, ~30 min, feedback), (c) feedback session 2 (~7 days after baseline, ~30 min, feedback), (d) post-feedback session 2 (directly after feedback session 2, ~30 min, no feedback), (e) follow-up 1 (~1 week after feedback session 2, ~45 min, no feedback), (f) follow-up 2 (~3 weeks after feedback session 2, ~45 min, no feedback). Feedback condition duration: about 60 min
Owlia et al. [70]	Directly after (≤4 h) ^7^	Order: Day 1 (1 h): (a) trial 1 (~10 min, no feedback: intervention group and control group), (b) video training (intervention group only), (c) trial 2 (~10 min, no feedback: intervention group and control group), (d) trials 3 and 4 (each ~10 min, feedback: intervention group, no feedback: control group). Day 2 (1 h): (e) trial 5 (~10 min, no feedback: intervention group and control group), (f) trials 6 and 7 (each ~10 min, feedback: intervention group, no feedback: control group), (g) trial 8 (no feedback: intervention group and control group). Feedback condition duration: about 40 min ^5^
Ribeiro et al. [72]	During feedback Very short term (≤1 week) Short term (≤1 month) Midterm (≤3 months) Long term (≥ 6 to <12 months) Very long term (≥ 12 months)	Order: (a) baseline, (b) intervention (4 weeks), (c) follow-up (after 1 week, 1 month, 3 months, 6 months, and 12 months). Feedback condition duration: 4 work weeks

Notes: ^1^ Hwang, Jaejin. 2024. e-mail message to author, January 5; ^2^ data recorded the last six minutes of each condition; ^3^ this includes visual feedback and note taking, in addition to auditory and vibration feedback; ^4^ the effect while receiving the feedback (i.e., during feedback) was also reported, but was not evaluated statistically, therefore excluded here; ^5^ Dutta, Talik. 2024. e-mail message to author, January 21; ^6^ performed in the participants real work tasks, hence no training session; ^7^ the effect while receiving the feedback (i.e., during feedback) was also reported, but was not evaluated statistically, therefore excluded here; ^8^ feedback provided half of the time the criteria were fulfilled: ^9^ excluded in the analysis of this review.

**Table 9:** Summary of the feedback characteristics and trigger.

Study	Feedback Type	Feedback Initiation	Feedback Modality	Feedback Timing	Feedback Trigger
Ailneni et al. [84]	Corrective	System determined	Vibration	Concurrent (cumulative)	One body region (neck) and one feedback level: neck flexion/inclination angle >15° occurring >30 s.
Bazazan et al. [85]	Corrective	System determined	Vibration or auditory ^1^	Concurrent ^2^	One body region (back) and one feedback level: Slouching trunk posture ^3^.
Boocock et al. [86]	Corrective	System determined	Auditory	Concurrent	One body region (back) and one feedback level: lumbosacral range of motion >80% of maximum.
Bootsman et al. [87]	Corrective	System determined	Auditory and vibration (condition 1) Auditory, vibration, and visual (condition 2)	Concurrent (cumulative)	One body region (back) and one feedback level: lower back flexion >20° for >1.5 s. Not more than 1 notification per 5 min.
Doss et al. [74]	Corrective ^4^	System determined	Auditory ^5^	Concurrent	One body region (back) and one feedback level: trunk flexion >45°.
Kamachi et al. [79]	Corrective	System determined	Auditory	Concurrent and fading	One body region (back) and two feedback levels: forward lumbar flexion 20° less than 70% maximum (intermittent tone), forward lumbar flexion >70% of maximum (continuous tone). Two variations of fading feedback: (a) feedback provided each time criteria were fulfilled (i.e., 100%), (b) feedback provided half of the time the criteria were fulfilled (i.e., 50%).
Kuo et al. [73]	Corrective	System determined	Vibration	Concurrent (cumulative)	One body region (back/neck) and one feedback level: slouching posture (head, neck, and upper trunk).
Langenskiöld et al. [76]	Corrective and reinforcing ^6^	System determined	Vibration	Terminal	Two body regions (arm and back) and one feedback level each: trunk inclination >30° occurring >10% of the time, arm elevation >30° occurring >30% of the time.
Lim et al. [75]	Corrective	System determined	Vibration	Concurrent (cumulative)	One body region (back) and two feedback levels: trunk inclination >45° (3 intermittent vibrations), if the vibration was triggered >2 times within 2 min (3 s continuous vibration).
Lind et al. [71]	Corrective	System determined	Vibration	Concurrent	Two body regions (arm and back) and two feedback levels each (lower versus higher vibration intensity): arm elevation ≥30°, arm elevation ≥60°, trunk inclination ≥20°, trunk inclination ≥45°.
Lind et al. [78]	Corrective	System determined	Vibration	Concurrent	One body region (arm) and two feedback levels (lower versus higher vibration intensity): arm elevation ≥30°, arm elevation ≥60°.
Lind et al. [77]	Corrective	System determined	Vibration	Concurrent	One body region (back) and two feedback levels: trunk inclination >30° (intermittent vibration), trunk inclination >45° (continuous vibration).
Owlia et al. [70]	Corrective	System determined	Auditory	Concurrent	One body region (back) and two feedback levels: forward lumbar flexion 20° less than 70% maximum (intermittent audible tone), forward lumbar flexion >70% of maximum (continuous audible tone).
Ribeiro et al. [72]	Corrective	System determined	Auditory	Concurrent (cumulative)	One body region (back) and two feedback levels: condition 1: lumbopelvic forward bending ≥45° occurring continuous >5 s; condition 2: lumbopelvic forward bending ≥45° occurring within 25 s after condition 1.

Notes: ^1^ most used vibration (Bazazan, Ahmad. 2024. e-mail message to author, January 31); ^2^ Bazazan, Ahmad. 2024. e-mail message to author, January 31. ^3^ a warning continued until the operator corrected the participant’s awkward shoulder posture from a slouched position to an upright trunk position (Bazazan, Ahmad. 2024. e-mail message to author, January 31); ^4^ also verbal instructions from coach if needed; ^5^ auditory feedback from system and verbal feedback from coach after each trial; ^6^ corrective (group A), corrective and reinforcing feedback (group B).

**Table 10:** Summary of the instruments for collecting and analyzing the motion data.

Study	Equipment for Analyzing the Exposure and Triggering the Feedback	Motion Sensor, Location and Attachment/Position Acc = Triaxial Accelerometers	Feedback Device, Location and Attachment/Position	Wearable
Ailneni et al. [84]	Commercial system Alex (NAMUInc., Seoul, South Korea). Smartphone Android application Bluetooth	One Acc (Alex ^1^) Neck: posterior side Location: posterior side of the neck Attachment: secured bilaterally around the ears	Commercial system: smartphone and smartphone application Location: posterior side of the neck Attachment: secured bilaterally around the ears	Wearable
Bazazan et al. [85]	Custom	Motion sensor: not reported Location: the back Attachment: straps around the shoulders	Custom system: smartphone and smartphone application Trunk (back side) Straps around the shoulders	Wearable
Boocock et al. [86]	Custom Custom-designed software that was run off a PC (built in LabView)	Two IMUs ^2^ (Shimmer Sensing, Ireland) Location: (backside) at first lumbar spinous process and at the sacral body (S1) Attachment: fixed to the skin	Custom system: custom-designed software that was run off a PC and built in LabView. Feedback from external in the room ^2^	Partly wearable
Bootsman et al. [87]	Custom Smartphone Android application Bluetooth communication	Two IMUs (LSM9DSO, STMicroelectronics, Sweden) Location: (back) lumbar spine vertebrae (L1 and L5) Attachment: in customized tight-fitting shirt	Custom system: smartphone and smartphone application Location and attachment: NR	Wearable
Doss et al. [74]	Custom Smartphone Android application (PostureCoach, Toronto Canada),	Two accelerometer-based sensors (Shimmer, Dublin, Ireland) Location: (back) thoracic vertebrae (T3–T4) and lower back (L5–S1) Attachment: custom made vest-like and belt-like harness and secured with Velcro tape	Custom system: PostureCoach ^1^ smartphone and smartphone application Location and attachment: NR	Partly wearable
Kamachi et al. [79]	Custom PostureCoach v0.2 Not wireless (cables)	Two IMUs (MTi-3, Xsens Technologies, Enschede, Netherlands) Location and attachment: (back) thoracic vertebrae (T10) using adjustable straps, and (back) approx. to sacrum and secured with a sacroiliac belt.	Custom system: PostureCoach ^1^ Located at waist height with a sacroiliac belt.	Wearable
Kuo et al. [73]	Commercial system: Lumo Lift, Lumo Bodytech Inc., Palo Alto, CA, USA) Smartphone Android application Bluetooth communication	One Acc (Lumo Lift ^1^) Location: below the clavicle, and midway between the sternal notch and the acromion process. Attachment: on the skin	Commercial system: Location and attachment: same as motion sensor	Wearable
Langenskiöld et al. [76]	Custom Smartphone Android application (ErgoRiskLogger ^3^) Bluetooth communication	Two IMUs (LPMS-B2 IMU, LP Research, Tokyo, Japan) Location: (back) about at the level of 1–2 thoracic vertebrae, and distal part of m. deltoideus Attachment: inside customized pockets of a tight stretchy workwear t-shirt	Custom system: the Smart Workwear System ^1^ Location: chest (upper part) and distal part of m. deltoideus Attachment: in customized pockets of a tight stretchy workwear t-shirt	Wearable
Lim et al. [75]	Custom Bluetooth 4.2 Raspberry Pi 3 board and PC	Four IMUs (Mbientlab MetaMotionR+) Location: at sixth thoracic vertebra, right thigh, right shin, and dominant wrist Attachment: to the skin using hypoallergenic double-sided tape	Custom system Location: back at sixth thoracic vertebra and dominant wrist Attachment: to the skin using hypoallergenic double-sided tape	Partly wearable
Lind et al. [71]	Custom Smartphone Android application (ErgoRiskLogger ^3^) Bluetooth communication	Two IMUs (LPMS-B2 IMU, LP Research, Tokyo, Japan) Location: (back) about at the level of 1–2 thoracic vertebrae, and distal part of m. deltoideus Attachment: inside customized pockets of a tight stretchy workwear t-shirt	Custom system: the Smart Workwear System^1^ Location: chest (upper part) and distal part of m. deltoideus Attachment: in pockets of customized straps	Wearable
Lind et al. [78]	Custom Smartphone Android application (ErgoRiskLogger ^3^) Bluetooth	One IMU (LPMS-B2 IMU, LP Research, Tokyo, Japan) Location: distal part of m. deltoideus Attachment: inside a customized pocket of a tight stretchy workwear t-shirt	Custom system: the Smart Workwear System ^1^ Location: distal part of m. deltoideus Attachment: in a pocket of a customized strap	Wearable
Lind et al. [77]	Custom Smartphone Android application (ErgoRiskLogger ^3^) Bluetooth	One IMU (LPMS-B2 IMU, LP Research, Tokyo, Japan) Location: (back) about at the level of 1–2 thoracic vertebrae Attachment: inside a customized pocket of a tight stretchy workwear t-shirt	Custom system: the Smart Workwear System ^1^ Location: chest (upper part) Attachment: in customized pockets of a tight stretchy workwear t-shirt	Wearable
Owlia et al. [70]	Custom PostureCoach v0.2 Not wireless (cables)	Two IMUs (MTi-3, Xsens Technologies, Enschede, Netherlands) Location and attachment: (back) approx. 10th thoracic vertebrae using adjustable straps, and back approx. at the sacrum secured with a sacroiliac belt.	Custom system: PostureCoach ^1^ Location: around the hip (lateral position) Attachments: waistband	Wearable
Ribeiro et al. [72]	Commercial system: Spineangel (Movement Metrics Ltd., Hamilton, New Zealand)	One Acc (Spineangel ^1^) Location: around the hip (lateral position) Attachments: belt or waistband	Commercial system: (Spineangel ^1^) Location: around the hip (lateral position) Attachments: belt or waistband	Wearable

Notes: Acc: triaxial accelerometers; ^1^ see more information on the system/instruments in column: Equipment for analyzing the exposure and trigger the feed-back; ^2^ Boocock, Mark. 2024. e-mail message to author, February 1; ^3^ [78,89].

**Table 11:** Results from high-quality studies evaluating the impact of augmented feedback in controlled work environments. Significant decreases in exposure are indicated by green, non-significant decreases by yellow, and non-significant increases by orange. *p*-values are shown in parentheses.

Study	Reported Effect from Feedback			
	Group mean (absolute) difference of max lumbosacral flexion (feedback group vs. control group) ^8^			
Boocock et al. [86]	During feedback administration			
	Lumbosacral flexion angle: ↓8% ^1,2^ (0.033) ^3^		Trunk flexion angle: ↓18.6% ^1,2^ (0.004) ^3^	
Kamachi et al. [79]	Group mean difference in distribution of lumbar spine flexion angle (intervention group vs. control group)			
	Effect directly after (≤4 h) feedback administration			
	Caregiving task 80th ↓17% (0.012) 95th ↓15% (0.036)			
	Short term, (≤1 month) after feedback administration			
	Caregiving task 80th ↓21% (0.001) 95th ↓23% (<0.001)		Skill transfer task 80th ↓ ^4^ (ns) 95th ↓ ^4^ (ns)	
	Midterm, (≤3 months) after feedback administration			
	Caregiving task 80th ↓14% (0.024) 95th ↓13% (0.024)		Skill transfer task 80th ↓ ^4^ (ns) 95th ↓ ^4^ (ns)	
Lim et al. [75]	Group mean difference in distribution of trunk flexion angle (feedback condition vs. baseline)			
	During feedback administration			
	Lifting-lowering task ^7^	Shoveling ^7^		Tying rebar ^7^
	back-position ^5^ 50th ↓38% (<0.05) 90th ↓18% (<0.05) 95th ↓14% (<0.05) wrist-position ^6^ 50th ↓48% (<0.05) 90th ↓21% (<0.05) 95th ↓15% (<0.05)	back-position ^5^ 50th ↓35% (<0.05) 90th ↓15% (<0.05) 95th ↓% ^4^ (ns) wrist-position ^6^ 50th ↓34% (<0.05) 90th ↓16% (<0.05) 95th ↓15% (<0.05)		back-position ^5^ 50th ↓% ^4^ (ns) 90th ↓% ^4^ (ns) 95th ↓% ^4^ (ns) wrist-position ^6^ 50th ↓% ^4^ (ns) 90th ↑% ^4^ (ns) 95th ↑% ^4^ (ns)
Lind et al. [71]	Median intra-individual differences in angle			
	Trunk inclination angle		Arm elevation angle	
	During feedback administration (first training session)			
	Cumulative time ≥20° ↓50% (0.003) ≥30° ↓50% (0.004) ≥45° ↓75% (0.007)	Distribution 50th ↓23% (0.002) 90th ↓31% (0.003) 99th ↓37% (0.006)	Cumulative time ≥20° ↓22% (ns) ≥30° ↑3% (ns) ≥45° ↑13% (ns)	Distribution 50th ↓5% (0.013) 90th ↓7% (0.004) 99th ↓3% (ns)
	During feedback administration (second training session)			
	Cumulative time ≥20° ↓55% (0.001) ≥30° ↓54% (0.002) ≥45° ↓92% (0.007)	Distribution 50th ↓31% (0.001) 90th ↓31% (0.001) 99th ↓37% (0.003)	Cumulative time ≥20° ↓30% (0.039) ≥30° ↓11% (0.042) ≥45° ↓4% (0.006)	Distribution 50th ↓10% (0.006) 90th ↓15% (0.002) 99th ↓9% (ns)
	Directly after (≤4 h) feedback administration (after first training session)			
	Cumulative time ≥20° ↓30% (0.001) ≥30° ↓35% (0.002) ≥45° ↓75% (0.005)	Distribution 50th ↓31% (0.001) 90th ↓12% (0.002) 99th ↓34% (0.003)	Cumulative time ≥20° ↓32% (0.033) ≥30° ↓19% (ns) ≥45° ↓4% (0.039)	Distribution 50th ↓10% (0.013) 90th ↓11% (0.004) 99th ↓7% (ns)

Notes: ns: not statistically significant; ^1^ absolute percentage; ^2^ the lumbosacral flexion angle and the trunk flexion angle increased for both groups at the 20th minute, but less in absolute percentage for the feedback group; ^3^ refers to the slope of the trend from the 1st to 20th minute; ^4^ no values reported, only visually in a figure; ^5^ vibration unit on back; ^6^ vibration unit on wrist; ^7^ no statistically significant differences between back and wrist position; ^8^ 20th minute compared to the 1st minute.

**Table 12:** Results from high-quality studies evaluating the impact of augmented feedback in controlled environments. Significant decreases in exposure are indicated by green, non-significant decreases by yellow, and non-significant increases by orange. *p*-values are shown in parentheses.

Study	Reported Effect from Feedback			
Ailneni et al. [84]	Group mean difference (feedback condition vs. control condition)			
	During feedback administration			
	Sitting workstation Neck flexion: ↓6°, ↓9% (0.002) Head inclination: ↓2°, ↓2% (0.156) Neck moment: ↓0.4 Nm, ↓13% (0.028)		Standing workstation Neck flexion: ↓5°, ↓7% (<0.0001) Head inclination: ↓3°, ↓4% (0.038) Neck moment: ↓0.5 Nm, ↓15% (<0.0001)	
Doss et al. [74]	Group mean difference in peak trunk kinetics (feedback condition vs. baseline)			
	Directly after (≤4 h) feedback administration			
	Flexion angle Task 1 Sling: ↓4° (ns) Task 2 Bed: ↓7.6° (0.05) Task 3 Adjust: ↓2.1° (ns)		Velocity (°/s) Task 1 Sling: ↑ 8.8 °/s (ns) Task 2 Bed: ↓9.9°/s (sign) Task 3 Adjust: ↑ 3.7 °/s (ns)	Acceleration (°/s^2^) Task 1 Sling: ↑ 231°/s^2^ (ns) Task 2 Bed: ↓1548°/s^2^ (sign) Task 3 Adjust: ↓45°/s^2^ (ns)
Kuo et al. [73]	Group mean difference in angle (feedback condition vs. control condition)			
	During feedback administration			
	Head tilt: ↑5% (ns) Neck flexion: ↓5%(<0.001) Upper cervical: ↓2% (0.004) Lower cervical: ↓2% (0.012)		Thoracic: ↓6% (0.033) Lumbar: ↓10% (ns) Pelvic plane: ↓30% (0.021)	
Langenskiöld et al. [76]	Group mean difference (feedback condition vs. baseline)			
	Trunk inclination angle		Arm elevation angle	
	During feedback administration			
	Proportion of the time ≥20° ↓15% (ns) ≥30° ↓19% (0.026) ≥45° ↓36% (0.008)	Distribution 50th ↓41% (ns) 90th ↓12% (ns) 99th ↓9% (ns)	Proportion of the time ≥30° ↓11% (ns) ≥45° ↓18% (0.008) ≥60° ↓20% (0.002)	Distribution 50th ↓9% (0.016) 90th ↓8% (0.003) 99th ↓3% (ns)
	Directly after (≤4 h) feedback administration			
	Proportion of the time ≥20° ↓23% (0.028) ≥30° ↓27% (0.014) ≥45° ↓54% (0.008)	Distribution 50th ↓94% (ns) 90th ↓18% (0.012) 99th ↓19% (0.008)	Proportion of the time ≥30° ↓10% (0.019) ≥45° ↓15% (0.002) ≥60° ↓17% (0.001)	Distribution 50th ↓8% (0.002) 90th ↓8% (<0.001) 99th ↓5% (ns)
Lind et al. [78]	Group mean difference in arm elevation (feedback condition vs. baseline)			
	During feedback administration			
	Feedback training (first session)		Feedback training (second session)	
	Proportion of the time ≥30° ↓38% (<0.001) ≥45° ↓36% (<0.001) ≥60° ↓49% (0.001)	Distribution 50th ↓32% (<0.001) 90th ↓16% (<0.001) 95th ↓10% (0.002) 99th ↓13% (0.001)	Proportion of the time ≥30° ↓38% (<0.001) ≥45° ↓45% (<0.001) ≥60° ↓65% (<0.001)	Distribution 50th ↓33% (<0.001) 90th ↓21% (0.001) 95th ↓19% (0.001) 99th ↓16% (<0.001)
Owlia et al. [70]	Difference in the distribution of lumbar spine flexion (feedback condition vs. baseline)			
	Directly after (≤4 h) feedback administration			
	Control group 50th ↓ *(ns) 80th ↑ *(ns) 95th ↑ *(ns)		Intervention group 50th ↓ *(ns) 80th ↓36% ^†^ (0.024 ^‡^) 95th ↓29% ^†^ (0.002)	

Notes: * no values reported (only visually in figure); † a part of this reduction may be related to the video training; ‡ reported (incorrectly) as 0.24 in the manuscript (Dutta, Talik. 2024. e-mail message to author, January 21).

**Table 13:** Summary of the consistency of the evidence for the effectiveness of feedback in controlled environments.

Study	Feedback Follow-Up Duration			
	During Feedback	Directly after (≤4 h)	Short Term (≤1 Month)	Midterm (≤3 Months)
**High quality**				
Boocock et al. [86]	++			
Kamachi et al. [79]		++	+/=	+/=
Lim et al. [75]	+			
Lind et al. [71]	++	++		
**Moderate quality**				
Ailneni et al. [84]	++			
Doss et al. [74]		+/=		
Kuo et al. [73]	++			
Langenskiöld et al. [76]	+	++		
Lind et al. [78]	++			
Owlia et al. [70]		+/=		

Notes: ++ overall, consistent statistically significant findings indicate that feedback reduces postural exposure; + overall, statistically significant findings indicate that feedback reduces postural exposure; +/= mixed results with some significant findings indicating that feedback reduces postural exposure and insignificant results in one or both directions.

**Table 14:** Results from high-quality studies evaluating the impact of augmented feedback in real work environments. Significant decreases in exposure are indicated by green, non-significant decreases by yellow, and non-significant increases by orange. *p*-values are shown in parentheses.

Study	Reported Effect from Feedback	
Lind et al. [77] 2023	Median intra-individual differences in trunk inclination (feedback condition vs. baseline)	
	During feedback administration (first occasion)	
	Proportion of the time ≥30° ↓13% (ns) ≥45° ↓34% (0.015) ≥60° ↓80% (0.026)	Distribution (angle) 90th ↓6.0% (ns) 95th ↓17% (0.026) 99th ↓11% (0.033) 10th–90th ↓7.9% (0.011)
	During feedback administration (second occasion)	
	Proportion of the time ≥30° ↓68% (0.001) ≥45° ↓80% (<0.001) ≥60° ↓89% (0.001)	Distribution (angle) 90th ↓34% (0.002) 95th ↓29% (<0.001) 99th ↓36% (<0.001) 10th–90th ↓31% (<0.001)
	Directly after (≤4 h) feedback administration	
	Proportion of the time ≥30° ↓60% (<0.001) ≥45° ↓61% (0.002) ≥60° ↓67% (0.034)	Distribution (angle) 90th ↓34% (0.002) 95th ↓31% (0.001) 99th ↓23% (0.003) 10th–90th ↓31% (<0.001)
	Very short term, (≤1 week) after feedback administration	
	Proportion of the time ≥30° ↓15% (ns) ≥45° ↓3.4% (ns) ≥60° ↓4.6% (ns)	Proportion of the time 90th ↓12% (ns) 95th ↓13% (ns) 99th ↑1.7% (ns) 10th–90th ↓2.4% (ns)
	Short term, (≤1 month) after feedback administration	
	Proportion of the time ≥30° ↓7% (ns) ≥45° ↓33% (ns) ≥60° ↓44% (ns)	Distribution (angle) 90th ↓5.5% (ns) 95th ↓10% (ns) 99th ↓11% (ns) 10th–90th ↑0.1% (ns)
Ribeiro et al. [72]	Group mean difference in frequency exceeding lumbar postural threshold compared to baseline	
	Control group	Intervention group
	During feedback administration	
	↓0 .3 times/h, ↓3.4 %	↓0 .6 times/h, ↓8 %
	Very short term, (≤1 week) after feedback administration	
	↓0 .6 times/h, ↓8 %	↓0 .4 times/h, ↓5.9 %
	Short term, (≤1 month) after feedback administration	
	↓2 .2 times/h, ↓30 %	↓1.0 times/h, ↓15 %
	Midterm, (≤3 months) after feedback administration	
	↑0 .4 times/h, ↑5.5 %	↑3.3 times/h, ↑49 %
	Long term, (<12 months) after feedback administration	
	↑2 .1 times/h, ↑29 %	↑0 .6 times/h, ↑9 %
	Very long term, (≥12 months) after feedback administration	
	↓1.2 times/h, ↓16 %	↓1.4 times/h, ↓21 %

**Table 15:** Results from moderate-quality studies evaluating the impact of augmented feedback in real work environments. Significant decreases in exposure are indicated by green, and non-significant decreases by yellow. *p*-values are shown in parentheses.

Study	Reported Effect from Feedback	
Bazazan et al. [85]	Group mean difference in RULA score (feedback group vs. control group) *	
	Midterm, (≤3 months) after feedback administration	
	Neck: ↓0.4 (<0.05)	Trunk: ↓0.7 (<0.001)
	Long term, (<12 months) after feedback administration	
	Neck: ↓0.3 (ns)	Trunk: ↓0.7 (<0.001)
Bootsman et al. [87]	Group mean difference of poor posture episodes (feedback condition vs. baseline)	
	During feedback administration	
	1st time: frequency ↓13.5% (sign ^†^)	2nd time ^‡^: frequency ↓25.3% (sign ^†^)
	Directly after (≤4 h) feedback administration	
	After 1st time feedback: frequency ↓2.7% (ns)	

Notes: * no stat. sign. difference at baseline before the feedback was provided; ^†^ the sign level was not reported; ^‡^ also included visual feedback with note-taking.

**Table 16:** Summary of the consistency of the evidence for the effectiveness of feedback in real work environments.

Study	Feedback Follow-Up Duration						
	During Feedback	Directly after (≤4 h)	Very Short Term (≤1 Week)	Short Term (≤1 Month)	Midterm (≤3 Months)	Long Term (≥ 6 to <12 Months)	Very Long Term (≥ 12 Months)
**High quality**							
Lind et al. [77]	++	++	(+)/=	(+)/=			
Ribeiro et al. [72]	=		=	=	=	=	=
**Moderate quality**							
Bazazan et al. [85]					++	+/=	
Bootsman et al. [87]	++	(+)/=					

Notes: ++ overall, consistent statistically significant findings indicate that feedback reduces postural exposure; +/= mixed results with some significant findings indicating that feedback reduces postural exposure and insignificant results in one or both directions; (+)/= overall, insignificant results of a tendency indicating that feedback reduces postural exposure; = overall, insignificant results in both directions.

## Data Availability

Not applicable. Database searches were conducted only for this review.

## Appendix A. Search Strings

**Table A1:** The search strings used in the literature search in Web of Science and Medline on 30 November 2023.

“feedback” or “biofeedback” AND “posture$” or “postural” or “movement$” AND “neck” or “trunk” or “spine” or “upper back” or “lower back” or “arm$” or “wrist$” The following terms were excluded “gait”, “child*”, “rehab*”, “parkinson*”, “*stroke*”, “cerebral palsy”, “spinal cord injury”, “prosthesis”, and “therap*”. Review articles were excluded. The search terms were applied to the abstract of each source, and covered the period 1 January 2020 to 30 November 2023.

**Table A2:** The search strings used in the literature search in Embase on 1 December 2023.

“feedback” or “biofeedback” AND “posture” or “postures” “postural” or “movements” AND “neck” or “trunk” or “spine” or “upper back” or “lower back” or “arm” or “arms” or “wrist” or “wrists” The following terms were excluded: “parkinson”, “stroke”, “child”, “cerebral palsy”, “spinal cord injury”, “prosthesis”, “therap*”, and “cadaver”. Review articles were excluded. The search terms were applied to the abstract, title and keywords of each source, and covered the period 1 January 2020 to 1 December 2023.

## Appendix B. The Criteria Used to Assess the Methodological Quality

**Table A3:** The criteria used to assess the methodological quality of observational cohort and cross-sectional studies.

Item	Criteria
1. Research question/objective clearly stated	The research question or objective of the study was clearly stated, and the outcome (dependent variable) was described at least on a general level
2. Study population clearly specified and defined	Clearly defined study population and inclusion/exclusion criteria, and the inclusion/exclusion criteria were consistently applied to all participants
3. Participation rate of eligible persons ≥50%	The participation rate of eligible persons was ≥50% of the total identified pool of eligible persons
4. Subjects recruited from same/similar populations	All subjects were recruited from the same or similar populations, with uniformly applied inclusion/exclusion criteria
5. Sample size justification	The sample size is described and justified, ensuring it is sufficiently large to detect a difference in the main outcome with at least 80% power
6. Exposure(s) measured prior to outcome(s)	Exposures were measured before outcomes
7. Sufficient timeframe	The timeframe for feedback was described and was sufficient to induce behavioral changes
8. Dependent variable measured in category- or as continuous variable	The dependent variable (e.g., posture or movements) was assessed on a continuous or categorical scale
9. Independent variables clearly and measured appropriately	Relevant independent variables were controlled or measured, including the amount of work performed per time unit, and if the feedback trigger was clearly defined and consistently implemented
10. Dependent variable(s) assessed more than once	The dependent variable(s) was assessed more than once
11. Dependent variable clearly defined and adequately assessed	The dependent variable was clearly defined and adequately assessed
12. Blinding of assessors	The assessors were blinded to the participants’ group assignment.
13. Loss to follow-up after baseline of ≤20%	The loss to follow-up after baseline is no more than 20%, ensuring data from at least 80% of participants are included in the final analysis
14. Adjusted for key confounding variables	The study adjusts for key confounding variables that could alter the outcome results

**Table A4:** The criteria used to assess the methodological quality of controlled intervention studies.

Item	Criteria
1. Study description, randomized RCT	The study was described as a randomized controlled trial and provides adequate details about the study design
2. Adequate method of randomization	A suitable randomization method was used, e.g., computer-generated random assignment of participants
3. Concealed treatment allocation	The process of assigning participants to group (e.g., intervention versus control) was concealed
4. Providers and participants blinded	Both those administering the intervention and the participants receiving it were blinded to group assignment
5. Assessors blinded the participants	Assessors evaluating outcomes were blinded to group assignment
6. Baseline characteristics that could affect outcomes	Baseline characteristics, such as age, gender, experience, occupation, job exposure, and disorders were reported and balanced between groups
7. Endpoint dropout rate of ≤20%	The dropout rate at the end of the study was ≤20%
8. Endpoint dropout rate between treatment groups of ≤15%	The dropout rate between groups at the end of the study was ≤15%
9. High adherence to intervention protocols in each group	Participants in each group adhered to the intervention protocol
10. Other interventions avoided or similar in the group	Participants did not receive additional interventions that could confound the study results, or any such interventions were similar between groups
11. Outcomes assessed using valid and reliable measures	The outcomes of the study were measured using accurate and precise tools/methods
12. Sample size justification	The sample size was described and justified, ensuring it is sufficiently large to detect a difference in the main outcome between groups with at least 80% power
13. Prespecified analysis of outcomes reported	The analysis of the outcomes was specified a priori conducting the analysis
14. Randomized participants analyzed in original group	Participants were analyzed based on their original group assignment
